# Distribution and diversity of fungi in freshwater sediments on a river catchment scale

**DOI:** 10.3389/fmicb.2015.00329

**Published:** 2015-04-21

**Authors:** Jie Liu, Jianan Wang, Guanghai Gao, Mark G. Bartlam, Yingying Wang

**Affiliations:** ^1^Key Laboratory of Pollution Processes and Environmental Criteria (Ministry of Education), Tianjin Key Laboratory of Environmental Remediation and Pollution Control, College of Environmental Science and Engineering, Nankai UniversityTianjin, China; ^2^Department of Environmental Science and Engineering, Nankai University Binhai CollegeTianjin, China; ^3^State Key Laboratory of Medicinal Chemical Biology and College of Life Sciences, Nankai UniversityTianjin, China

**Keywords:** fungal diversity, biogeography, river catchment, DGGE, redundancy analysis (RDA)

## Abstract

Fungal communities perform essential functions in biogeochemical cycles. However, knowledge of fungal community structural changes in river ecosystems is still very limited. In the present study, we combined culture-dependent and culture-independent methods to investigate fungal distribution and diversity in sediment on a regional scale in the Songhua River catchment, located in North-East Asia. A total of 147 samples over the whole river catchment were analyzed. The results showed that compared to the mainstream, the tributaries have a higher fungal community organization and culturable fungal concentration, but possess lower community dynamics as assessed by denaturing gradient gel electrophoresis (DGGE). Furthermore, phylogenetic analysis of DGGE bands showed that *Ascomycota* and *Basidiomycota* were the predominant community in the Songhua River catchment. Redundancy analysis revealed that longitude was the primary factor determining the variation of fungal community structure, and fungal biomass was mainly related to the total nutrient content. Our findings provide new insights into the characteristics of fungal community distribution in a temperate zone river at a regional scale, and demonstrate that fungal dispersal is restricted by geographical barriers in a whole river catchment.

## Introduction

Sediment and their attached microbes make a substantial contribution to the biogeochemical processes of river ecosystems (Rastogi et al., 2011; Sanchez-Andrea et al., 2011), such as nutrient transformations, energy flow, food web and self-purification (Gerbersdorf et al., 2011). Due to their valuable services in the ecosystem, changes in fungal assemblages could provide insight into the physicochemical assessment of river water quality and ecosystem health (Amaral-Zettler et al., 2008; Liu et al., 2011). Therefore, it is important to elucidate the mechanisms linking community diversity and processes over time and space in response to different environmental conditions (Hazard et al., 2013). Recently, studies aimed at understanding the dynamics of fungal communities were conducted, with the major goal being to acquire knowledge about what controls the distribution and abundance of the microbial community and how these communities change in response to their environmental gradients (Tiedje et al., 1999; Logue et al., 2011; Read et al., 2011). Consequently, the driving factors of microbial community, which maintain biodiversity on the earth, have mainly been discussed in terms of contemporary disturbances (environmental heterogeneity) and historical contingencies (geographical distance) (Green and Bohannan, 2006; Ge et al., 2008; Vanormelingen et al., 2008; Schauer et al., 2010; Lindstrom and Langenheder, 2012; Hazard et al., 2013; Wu et al., 2013).

To date, wide controversies exist about whether fungi display a biogeographic distribution signature and which are the main factors that shape the community (Van der Gucht et al., 2007; Patterson, 2009; Bissett et al., 2010). It was reported that local environmental variations are mainly driving the changes in microbial community composition and are more distinctive than the temporal ones (Kolukirik et al., 2011). Similarly, Olsen and co-workers also showed that eukaryotic community structure was more highly correlated with environmental factors than geographical distance around the South Shetland Islands, Antarctica (Olsen et al., 2013). However, it was also reported that pure spatial effects clearly overcame those of environmental effects, with the former explaining the much greater variation in species richness and community composition (Heino et al., 2010). Microbial communities were considered to exhibit non-random spatial biogeographic patterns and are even scale-dependent (Yergeau et al., 2010; Wang et al., 2013). Wu and co-workers observed that fungal diversity variation was mainly affected by historical geographic distance (i.e., location, including altitude) at a large regional scale (1000–4000 km), and contemporary environmental conditions (total potassium and total nitrogen) could explain the variation in fungal diversity on a small local scale (<1000 km) (Wu et al., 2013). Similarly it was concluded that arbuscular mycorrhizal fungi (AMF) community composition is significantly changed with the geographical distance at the regional scale (250 km), while environmental heterogeneity was the major factor in determining turnover of AMF taxa at the landscape scale (van der Gast et al., 2011). Hence, further investigation is needed to resolve such controversies.

As previously reported, spatial turnover in the composition of biological communities is governed by ecological drift, selection and dispersal (Stegen et al., 2013). When comparing the differences between contemporary environmental variables and historical geographical distance, the precondition is that the geographic sampling sites should be stochastic, homogeneous or at least spatially correlated so as to eliminate the “noise” brought by non-random and deterministic influences of the research area (Valentin-Vargas et al., 2012; Wang et al., 2013). However, most of the previous studies have ignored this potential and critical problem. In the study reported here, we sought to resolve the above problem by setting up a series of successive locations on a catchment scale in a temperate zone river, which flows through mountains and plains and covers many types of landscape, including headstream, hills, forest, agricultural and industrial land and metropolis. Secondly, as the Songhua River is formed from a merger of two sub-mainstreams which have their sources in different regional areas and from different directions, it meets the demands of both geographic homogeneity and variance.

The aim of the current study was: (1) to elucidate the biogeographic distribution of fungal concentration and diversity in the sediment of Songhua River catchment; (2) to identify the unique species in the Songhua River catchment; and (3) to analyse the impact of sediment environmental variables and geographical distance on the fungal community.

## Materials and methods

### Study sites

**Figure 1:**
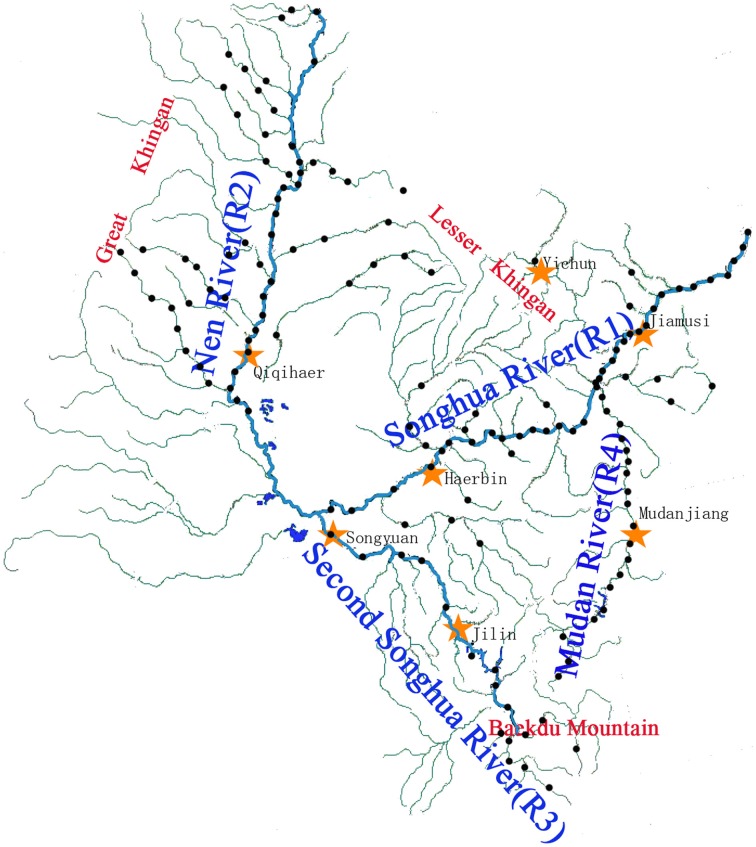
**Sampling site distribution map**. Two hundred forty-five sampling sites were set, as the sand and gravel in the river bottom zone, and 147 sediment samples were collected in the Songhua River catchment. Sampling was conducted from June to August in 2010. R1, the mainstreams of Songhua River; R2, the Nen River; R3, the second Songhua River; R4, the Mudan River.

The Songhua River is an important international wetland located in North-east Asia. All sampling and pretreatment of samples follow the China National Standards for Scientific Sampling (Ministry of Environmental Protection of China, 2009). The sediment samples were collected by a special sampling device which was manufactured by an environmental monitoring station in Harbin, Heilongjiang Province. Samples (a mixture of sediment from the upper 20 cm) were homogenized at sampling sites in clean glass containers. Samples were taken every 20 km along the mainstream and 40 km along the tributaries over the whole Songhua River Catchment (Figure 1). Sampling points were numbered and referenced using a global positioning system. A total of 147 samples were collected from June to August in 2010 before the rainy season. All samples were immediately sealed and stored at 4°C in 50 ml pre-cleaned centrifuge tubes during transportation.

### Heterotrophic plate counts

Enumeration and isolation of fungi was performed by the dilution plate method. Measurement of concentration of cultivable fungi employed potato dextrose agar (PDA) medium amended with chloromycetin (50 μg/ml) and streptomycin sulfate (50 μg/ml) to inhibit growth of bacteria and actinomyces. 2.00 g sediment was suspended in 18 ml sterile physiological saline, giving a 1:10 dilution; two successive dilutions (1:100 and 1:1000) were prepared to select a proper dilution gradient. 100 μL of suspension were spread on 90-mm-diameter plates containing PDA medium. Each suspension has three replicates. Plates were incubated at 28°C and recorded every 24 h until the highest number of colonies was reached after plating.

### PCR-denaturing gradient gel electrophoresis analysis

A freeze-thaw/phenol-chloroform extracting approach was applied to extract genomic DNA from sediment and 50 μl DNA solution was obtained from each sample. Then, the genomic DNA was purified by the E.Z.N.A.™ MicroElute DNA Clean-up Kit (Omega, USA). Nested PCR with primers sets ITS1-F/ITS4 (Gardes and Bruns, 1993) and ITS2/ITS1F-GC (White et al., 1990) was used to amplify fungal ITS? region. A 40 base GC clamp (5′-CGC CCG CCG CGC GCG GCG GGC GGG GCG GGG GCA CGG GGG G-3′) was attached to the 5′ end of the ITS1-F primer to stabilize the melting behavior of the DNA fragments during DGGE analysis (Sheffield et al., 1989). The first round of PCRs were carried out on an touchdown cycler using 25 μl reaction volumes containing: 1 μl DNA template, 0.25 μl 10 μM of each primers, 12.5 μl 2 × Go Taq® Master Mix(Promega, USA) and 11 μl nuclease-free water. PCR cycle was programed as follows: 94°C for 4 min followed by 10 cycles of 94°C for 1 min, lowering the annealing temperature from 65°C to 55°C at 1°C steps for each cycle for 1 min, 72°C for 1 min, and finally 25 cycles of 94°C for 1 min, 55°C for 1 min, 72°C for 1 min, and followed by finally extension at 72°C for 7 min. The second PCRs were carried out on a touchdown cycler using 50 μl reaction volumes containing: 1 μl first PCR production, 0.5 μl 10 μM of each primer, 25 μl 2 × Go Taq® Master Mix (Promega, USA) and 23 μl nuclease-free water. Cycling parameters were the same as the first round of PCR. All amplification products were electrophoresed in agarose gel 1.2% (w/v), stained with EB and visualized under UV light.

Denaturing gradient gel electrophoresis analysis was carried out using the Dcode™ universal mutation detection system (Bio-Rad, USA). Electrophoresis was performed on 8% polyacrylamide gels (acrylamide:bisacrylamide, 37.5:1) with a 15–45% denaturant agent vertical gradient (100% denaturants defined as 7 M urea and 40% (v/v) formamide. 20 μl PCR product mixed with 5 μl blue/orange loading dye (Promega, USA) was loaded onto the gels and the electrophoresis were run at 140 V and 60°C for 4 h in 7 L of 1 × TAE buffer (40 mM Tris, 20 mM acetic acid, 50 mM EDTA [pH 8.0]). The gels were stained for 30 min with 0.5 μg/ml EB and visualized with UV light using Molecular Imager ™ Gel Dox XR^+^ (Bio-Rad, USA).

Images of DGGE fingerprints were employed to quantify community structure using the Quantity One 4.31 software (Bio-RAD, USA) and Gelcompar II 6.5 (Applied Maths, Belgium) according to the manual of the provider, which detects bands and quantifies relative concentration of DNA. The DGGE pattern analysis was done using a previously reported protocol (Marzorati et al., 2008). The percentage change values for different adjacent sites were calculated based on the percent of similarity matrix values and was defined as the dynamics (Fromin et al., 2002; Pereira et al., 2010).

### Excising and sequencing interest DGGE bands

Individual DGGE bands were excised, re-suspended in 20 μl sterile TE buffer, and stored at 4°C overnight. An aliquot of supernatant was used as DNA template for PCR re-amplification as described above, and electrophoresed with DGGE. Band excision, PCR, and DGGE were repeated until a single band was present. For sequencing, PCR products generated from DGGE bands were amplified with primers ITS2 and ITS-1f (without the GC clamp). The sequences obtained were compared with Genbank database by using BLAST.

### Statistic analysis

Fungal distribution of abundance and diversity were drawn by Origin 8.0 software. A multivariate redundancy analysis (RDA) was performed by Canoco software (Canoco for Windows version 4.5, Microcomputer Power, Ithaca, USA) to illustrate the confounding changes among fungal abundance, communities structure and environmental factors (the content of organic matter, total phosphorus, quickly available phosphorus, total nitrogen, ammonia nitrogen, nitrate nitrogen, redox potential, pH, and samples location, including longitude, latitude and altitude). The diversity values of fungi were centered and standardized in the redundancy analysis, and environmental factors were also standardized as variables before performing RDA analysis. Meanwhile, the Monte Carlo test was used to examine the significance of analysis. A generalized linear model (GLM) and generalized additive model (GAM) was performed in a stepwise manner to predict the multiple response of fungal community to each variable factor, and the visualization formula was given in terms of linear, quadratic and cubic degree of GLM, and F statistics were conducted to test the significance of both GLM and GAM. ANOVAs analysis was used to assess the significance of differences in measured or calculated parameters. ANOVA analysis was performed using R statistical software (http://www.r-project.org/).

## Results

### Fungal community diversity distribution character at catchment scale

**Figure 2:**
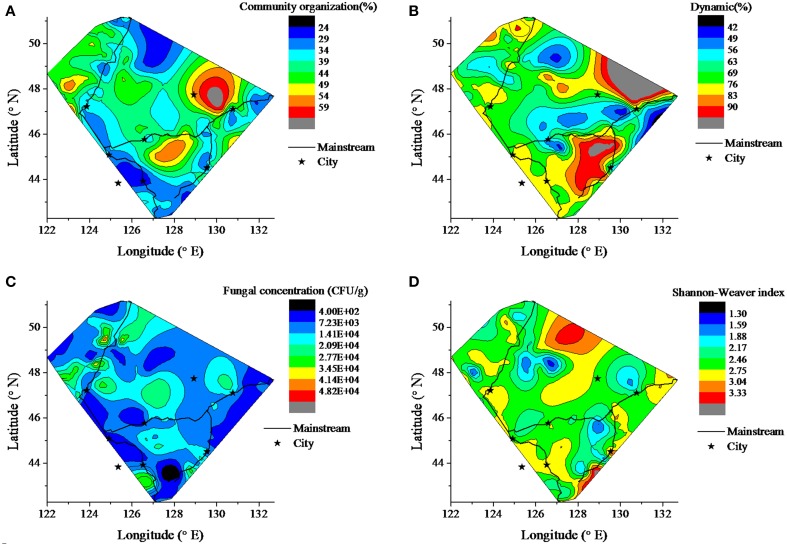
**Contour of fungal biodiversity in the sediment among the Songhua River catchment**. The map shows the fungal distribution of abundance and diversity information. **(A)** fungal community organization, **(B)** fungal dynamics, **(C)** fungal concentration, **(D)** Shannon Weaver index.

The contour of fungal community organization (Co), dynamic (Dy), diversity indices, including Shannon-Weaver index (HI), Simpson index (DI), Richness index (RI), and Evenness index (EI) and concentration (Cf) was drawn to illustrate the fungal geographic distribution in the sediment among the Songhua River catchment (Figure 2). Here, the community dynamics (Dy) is interpreted as the number of species that come to significant dominance and the community organization (Co) is defined as the functionality of the community to organize in an adequate distribution of dominant microorganisms and resilient ones, conditions that should assure the potentiality of counteracting the effects of a sudden perturbation exposure (Marzorati et al., 2008; Read et al., 2011). The sediment has significantly lower Cf in the river source region (headwater areas of R2 and R3) (*p* < 0.001). Compared to the mainstream, the tributaries have higher fungal community organization (Co) (*p* < 0.001) and culturable fungal concentration (Cf) (*p* < 0.001), but possess lower dynamics (*p* = 0.014). There is no significant difference in the Shannon-Weaver and richness index (data not shown) within the catchment.

**Figure 3:**
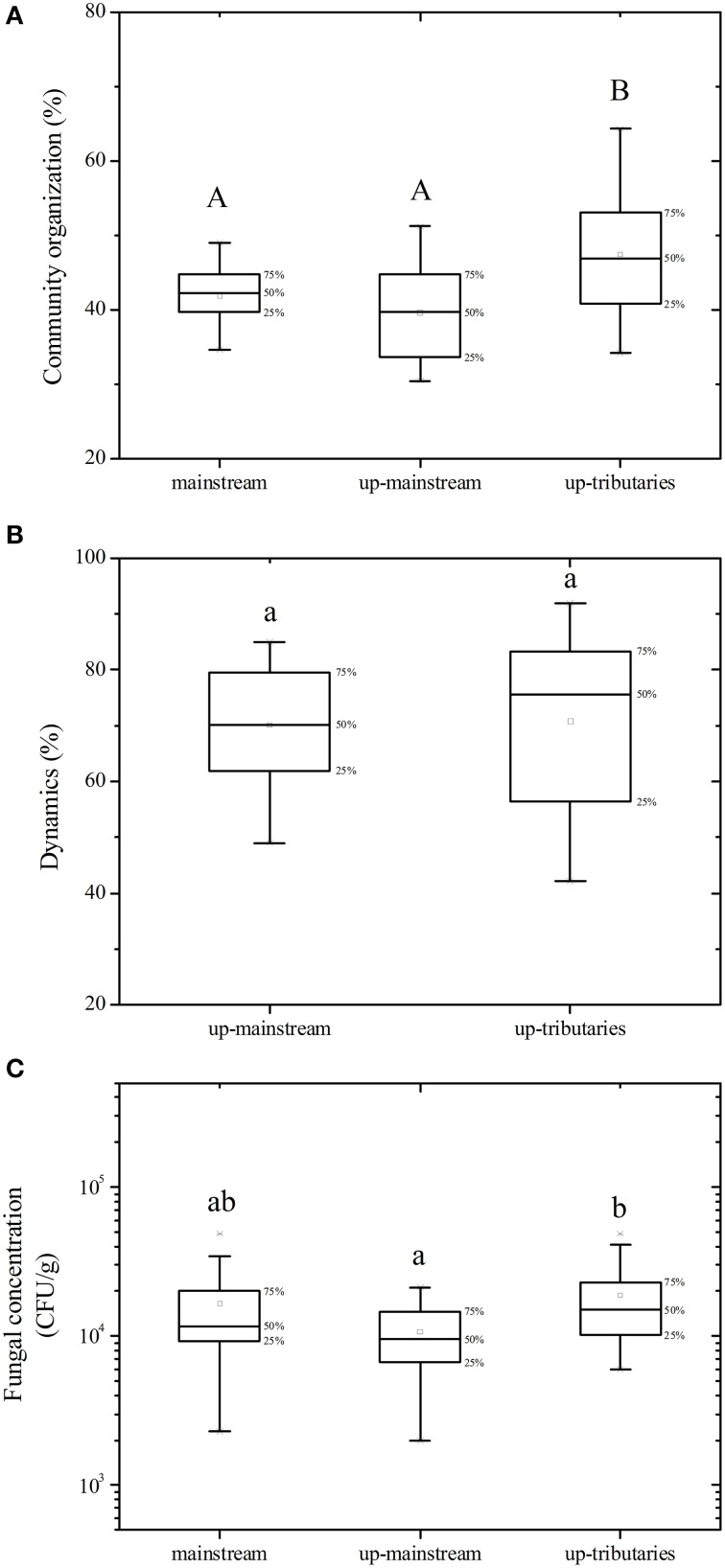
**Influence of the import from tributaries on the mainstream. (A)** fungal community organization, **(B)** fungal dynamics, **(C)** fungal concentration. Different letters in the same boxplot meant significant difference at 0.01 level (capital) and 0.05 level (lowercase) respectively.

A total of 16 estuaries were specifically analyzed to estimate the impact of the import from tributaries on the fungal community of the mainstream (Figure 3). The results show that the import of up-tributaries increased the concentration of fungi in the mainstream. Compared to the mainstream, the up-tributaries have a high fungal community organization (Co) (*p* = 0.005). Although no significant differences in dynamics values between mainstream to up-mainstream and mainstream to up-tributaries (*p* > 0.05) were observed, mainstream to up-tributaries have more dynamic variance (*p* = 0.033).

**Figure 4:**
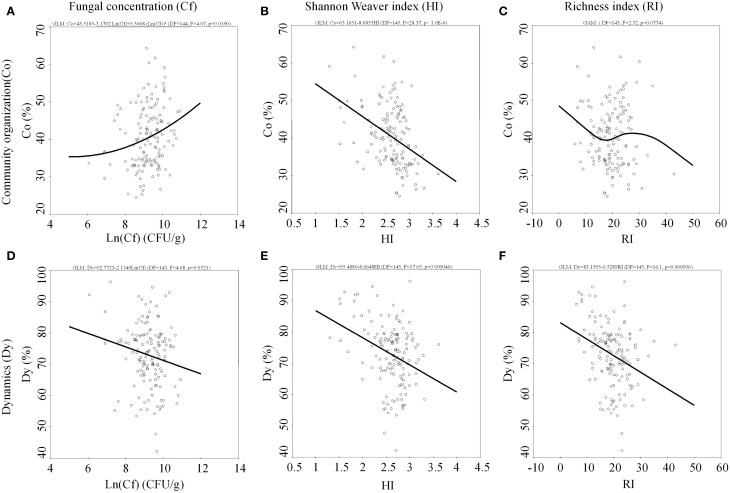
**Internal correlation of community organization to (A) fungal concentration, (B) Shannon Weaver index, (C) richness index and dynamics to (D) fungal concentration, (E) Shannon Weaver index, (F) richness index among the fungal diversity**.

In the present study, an interesting observation is that microbial community structure has an intrinsic correlation to community organization and dynamics in river ecosystem. Specifically, fungal community organization (Co) is positively correlated with the fungal concentration (*p* = 0.019) and negatively correlated with fungal Shannon-Weaver diversity (*p* < 0.001). There was a decreasing trend in terms of fungal richness (*p* = 0.0774) (Figure 4). The fungal dynamic (Dy) is negatively correlated with the fungal concentration (*p* = 0.0321), Shannon-Weaver diversity (*p* < 0.001) and richness (*p* < 0.001) in the Songhua river sediment.

### Phylogenetic analysis of fungal community

**Figure 5:**
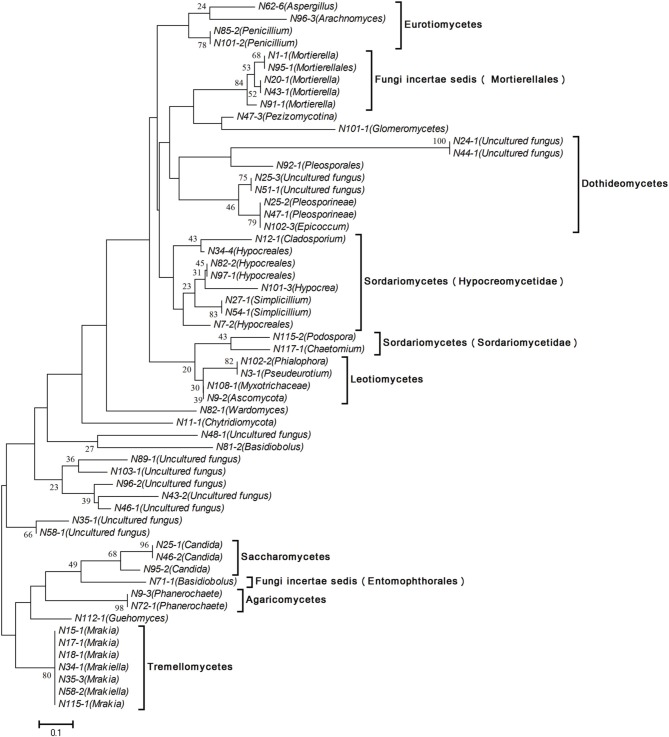
**Phylogenetic analysis of fungal ITS1-DNA sequences by means of the neighbor-joining method using genetic distances defined by Maximum Composite Likelihood**. The bar represents 10% sequence divergence. Numbers at the nodes represent bootstrap values 1000.

DGGE profiles from all fungi in Songhua River sediment revealed an obvious shift in fungal communities. Fifty eight bands were excised and further sequenced from the mainstream, including the common bands from all sample locations and the unique predominant bands from each sampling site. All entries are available from the European Nucleotide Archive (http://www.ebi.ac.uk/ena/data/view/LM655253-LM655310). The distance analysis was performed to provide more information and phylogenetic placement of these sequences (Figure 5). Partial bands showed the highest similarity, and FASTA analysis revealed those bands most closely related to the known species, whereas remaining one band sequences were not satisfactorily matched with any fungal sequences in the GenBank database and remained unknown. Meanwhile, the OUT proportion in each lane was calculated by quantitative analysis of the DGGE profile (Supplementary Material). Overall, the most dominant fungal genus was Ascomycota, followed by Basidiomycota and early diverging fungal lineages. In addition, 12 bands (20.69% of total bands) correspond to uncultured fungus (Figure 5).

The distribution of fungi showed a clear regional pattern. The sequence and phylogenetic analysis revealed each region within the catchment has its own individual common predominating species. Specifically, *Mrakia* was the common species in the mainstream of R1, *Myxotrichaceae* and Podospora were common species in mainstream of R2, and *Simplicillium* sp. and *Mrakia* sp. were common species in R4. However, the common predominant species in R3 and R4 were uncultured fungus and are still unknown. For instance, the proportion of uncultured fungus (band N51-1) reached 32.5% of the total fungi found in the N51 site (R3).

### Redundancy and response model analysis

**Figure 6:**
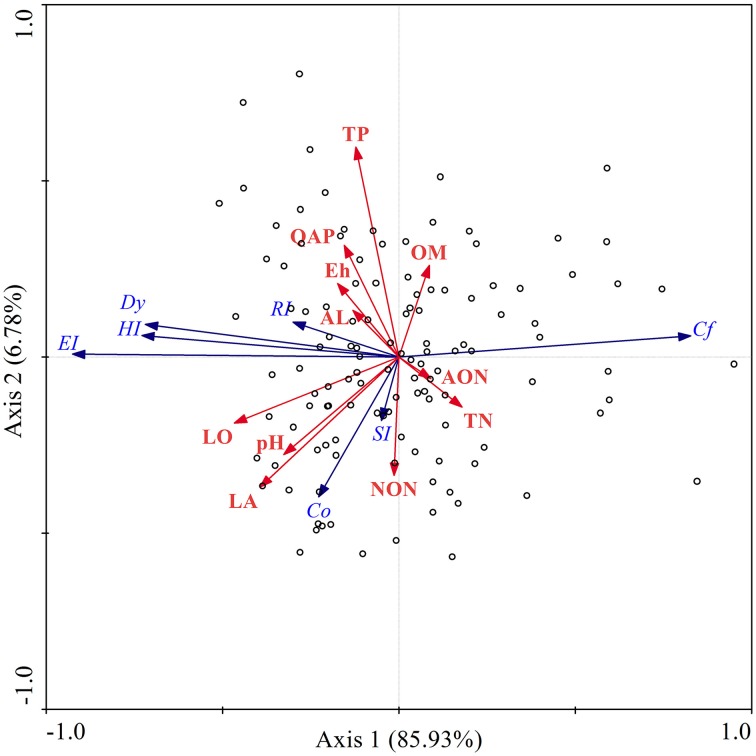
**Redundancy analysis (RDA) ordination biplot of sediment fungal abundance, biodiversity (blue arrows) and their respective physicochemical properties (red arrows) in the Songhua River catchment**. Co, community organization; Cf, fungal concentration; DI, Simpson index; Dy, community dynamic; EI, Evenness index; HI, Shannon-Weaver index; RI, Richness index; OM, organic matter; TP, total phosphorus, QAP, quickly available phosphorus; TN, total nitrogen; AON, ammonia nitrogen; NON, nitrate nitrogen; Eh, redox potential; LO, longitude; LA, latitude; AL, altitude; and pH.

The multivariate ordination technique is commonly used to interpret variation in the field of ecology, as it offers the greatest opportunity to combine statistical analysis of community profiles with various variables (Fromin et al., 2002). In the present study, redundancy analysis (RDA) was conducted to explain the changes in fungal community structure using environmental factors (Figure 6), where the first axis and second axis of RDA explained 85.93 and 6.78% of the variance, respectively. According to RDA, longitude was the most important factor to affect the fungal community changes, accounting for 19.82% community variation (*p* = 0.002), followed by total phosphorus (11.01%,), organic matter (11.01%,), quickly available phosphorus (11.01%,), total nitrogen (11.01%,), redox potential (8.81%,), ammonia nitrogen (8.81%,), altitude (8.81%,), nitrate nitrogen (6.61%,) and latitude (2.20%,). RDA analysis revealed that the variation of fungal community in the Songhua River catchment area was first interpreted by longitude, which implies that the variation is probably linked to the main direction of river flow, due to the flow direction being consistent with the longitudinally geographical gradients within the catchment area. The variation was then elucidated by nutrients (organic matter, total phosphorus, total nitrogen, quickly available phosphorus), where each parameter has the same weight of contribution to the variance of the fungal community (Supplementary Material). The results also reveal that the type of nitrogen elements exerts different degrees of effects on the fungal structure (Supplementary Material).

**Figure 7:**
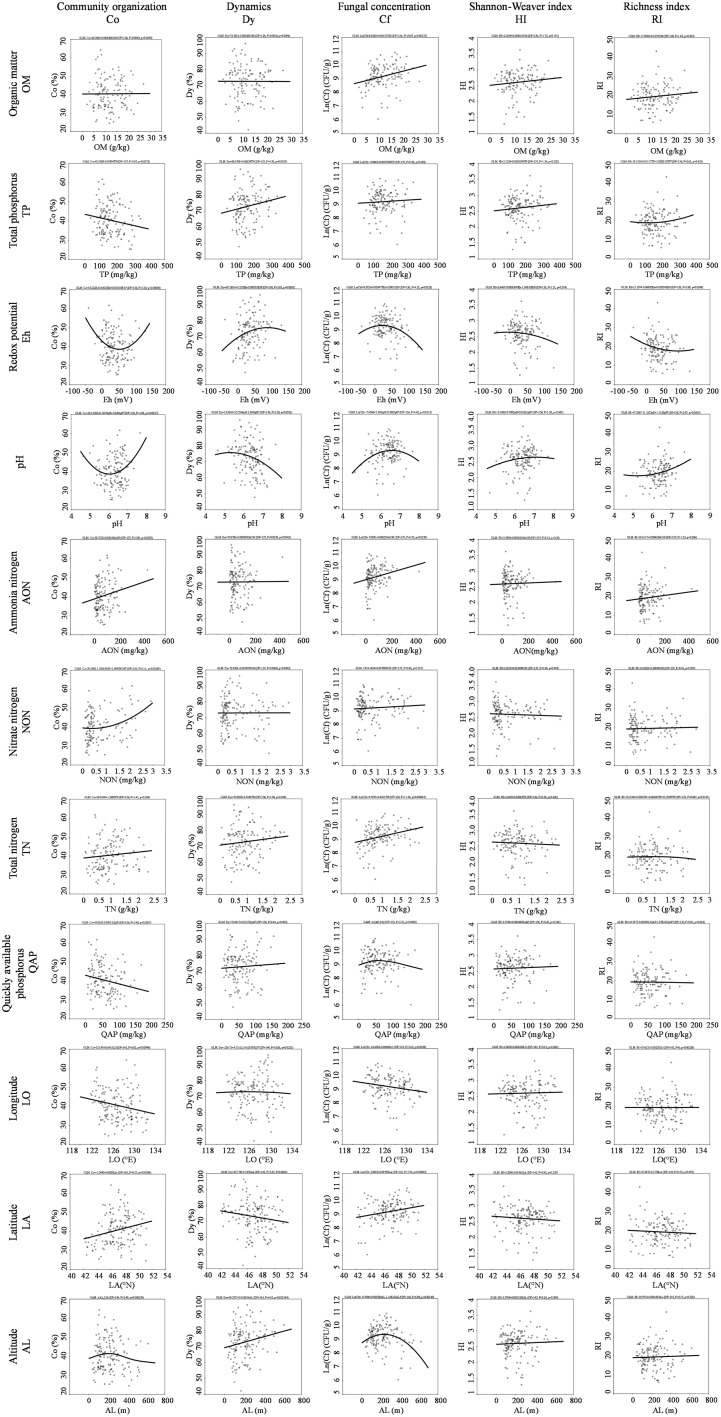
**Multiple fungal diversity index response curves to the environmental variable and geographic distance**.

Two response models, the generalized linear model (GLM) and generalized additive model (GAM), were applied to reveal detailed information for the multiple response of the fungal community to each variable factor. Fungal community organization, dynamic and concentration are well summarized via the visualization formula (Figure 7). The response model had a good fit to the fungal community structure, and demonstrated that fungal community was significant affected by geographical distance, including longitude, latitude and altitude. Co, Dy, and Cf decreased along the main direction of river flow (longitude: west to east) (Figure 7). In the low latitude area, the river sediment fungi has high Co and Cf, but low Dy. The fungal community possesses the highest Co and Cf at an altitude of ca. 230 m, though the fungal community becomes more dynamic with increasing altitude (Figure 7). The response model also revealed a close correlation between fungal community and various nutrients in river sediment, which indicates that the types of nutrient elements either restrict or promote fungal growth. Co was significantly correlated to the nitrate nitrogen, ammonia nitrogen, quickly available phosphorus and total P (Figure 7). Cf was significantly linked to organic matter, total N, and ammonia nitrogen (Figure 7). Eh and pH have a quadratic effect among Co, Dy, and Cf.

## Discussion

The question of whether microbes disperse globally remains controversial. In this study, the fungal community structure, composition and their response to extrinsic factors were investigated in the sediment of the whole Songhua River catchment at the succession and regional scale using both culture independent and dependent techniques. The distribution contour map and multivariate ordination was used to illustrate the biogeographical characteristics of the fungal community.

### Geographical patterns in fungal community

It was proposed that geographical barriers rarely restricted the dispersal of microbial eukaryotic abundance (Finlay, 2002), and physical characteristics of the habitats inordinately extend effects on surrounding communities and ecosystem function (Navel et al., 2012). However, the contours of the fungal community structure in study here, including the fungal community organization (Co), dynamic (Dy), and concentration (Cf), displayed a distinct geographical pattern between mainstream and tributaries (Figure 2). The headwater has a lower fungal concentration and, after flowing though the source region, the fungal concentration downstream was increased in the Songhua River. This phenomenon could be explained by the fact that nutrient concentration gradually increases from upstream to downstream due to nutrient transfer from terrestrial to aquatic systems via the processes of surface runoff, erosion and leaching (Peterson et al., 2001; Harner et al., 2004). The tributaries have greater fungal biomass (Cf) and more uneven distribution of fungal species abundance (Co). This is consistent with the theory that smaller streams (i.e., tributaries) have more effective nutrient transport and retention processes than big rivers (mainstream) (Peterson et al., 2001). The tributaries of the Songhua river derive from mountain forests, where vegetation has an indirect influence on the distribution, activity, and metabolic physiology of sediment microbial communities (Oliveira et al., 2010). After various tributaries merge into the mainstream, the fungal sediment habitat becomes more complex, and multiple factors could jointly control the composition and activity of the microbial community (Agnelli et al., 2004). Thus, many species become dominant, which leads to rapid changing and increasing dynamics of fungal community in the mainstream. As shown in Figure 3, the fungal community in the mainstream was more dynamic than in the tributaries. It has been reported that microbial diversity gradually changes from upstream to downstream along the Changjiang River (Sekiguchi et al., 2002). In the present study, when rivers flow through plains and residential areas, Cf and Co decreased in the downstream (Figure 2), which also reflects the great effect of land use and human activity on the fungal community (Sun et al., 2011; Gelorini et al., 2012).

In the study reported here, the intrinsic correlation of microbial community structure (see Figure 4) infers that stability of the fungal community (Co and Dy) does not necessarily imply a high fungal diversity (HI and RI), while microbial community functionality depends on the flexibility of the community structure, namely the ability of minority community members to become dominant in a short period under a sudden perturbation in conditions (Fernandez et al., 2000). The reason could be that a functional structural redundancy exists in the microbial community in nature (Bell et al., 2005). Similarly it was reported that medium values of Co (synonyms for Fo) were linked to high richness in the ammonia-oxidizing bacterial community (Wittebolle et al., 2009).

### Phylogenetic analysis

A clear and complex shift in DGGE pattern was found over the whole Songhua River catchment (Supplementary Material). Besides the common dominant fungi, almost each sampling point was found to have its individually predominant species, which also explains that fungal distribution was restricted by geographical locations. These common and uniquely predominant fungi possess special functions based on the local environmental ecosystem. Fungi can potentially indicate the degree of pollution stress. It has been reported that fermentative species are predominant populations in polluted water, and the abundance of yeasts is associated with the water quality in aquatic ecosystems (Medeiros et al., 2008). Dynowska's work showed that yeast-like fungi, such as *Candida* sp., *Rbodotorula* sp., *Cryptococcus* sp., and *Tricbosporon* sp., can be considered as bio-indicators of the progress in the process of eutrophication and accumulation of organic and inorganic pollutants (Dynowska, 1997). In the current study, the genus *Candida* (class of *Saccharomycetes)* is mainly represented in the river downstream of urban areas in R2 and R4 (Supplementary Material), which suggests that the river probably undergoes eutrophication in those areas due to anthropic activities. Our study reveals that numerous strains of genera *Mrakia/Mrakiella* and *Guehomycesare* (class of *Tremellomycetes*) are commonly predominant in R1 and R4 (Supplementary Material). Those fungi have previously been found in cold habitat regions and possess psychrophilic characteristics (Branda et al., 2010; Krishnan et al., 2011), possessing the strong ability to utilize nutrient and growth at low and even sub-zero temperatures (Thomas-Hall et al., 2010). The presence of *Tremellomycetes* confirms the local climate. The sampling location of the current study, the Songhua river catchment, is at the intersection between the temperate and cold-temperate zones and belongs to the terrestrial seasonal wind climate with an average annual temperature approximately between −1°C and 5°C (Sun et al., 2011). This peculiar low temperature climate condition may promote the formation and dominance of these cold-adapted yeast. Meanwhile, in the river catchment, we found a unique predominance of *Aspergillus* and *Penicillium* (class of *Trichocomaceae*), which could product beta-glucans, mycotoxins and surface proteins and increase the potential risk of health lesions (Houbraken and Samson, 2011). Furthermore, these genera can tolerate extreme environmental stress, e.g., low water availability and high temperatures (90°C), and can be recovered under appropriate conditions (McGee et al., 2006), which could enhance their ability to survive under various environmental conditions in the Songhua River. Based on the above discussion, the fungal community composition in the sediment of Songhua River was affected by the local climate (temperature) conditions and water eco-function in the catchment area.

### Impact of extrinsic factors on fungal communities

Although previous studies have shown that microbial diversity was mainly influenced by historical geographical distance, and that physical barriers have significant contributions to the fungal distribution (van der Gast et al., 2011; Wu et al., 2013). However, since those results were obtained from jumping sampling sites (distance between sampling sites is very large), one could not eliminate the “noise” brought about by the geographical gap in locations. In the current study, the conclusion was drawn from analysis of a large succession of sampling sites at medium scale (1000 km), which imbues the fungal community with geographic homogeneous and variance properties, and therefore it can avoid the gap noise. Here, our study confirms the theory for the first time on a continuous sampling scheme.

It was proposed that the microbial community exhibits a horizontal spatial distribution pattern that is correlated with latitude or climate gradient (Ge et al., 2008), which is based on a limited number of locations. In the current study, the changes in fungal community were independent of the latitudinal flow direction, and were different from community changes over longitude as fungal community variance was consistent with the longitudinal flow direction. Although the Songhua River is formed from a merger of two sub-mainstreams (R2 and R3 see in Figure 2) and the two sub-mainstreams have opposite latitudinal flow directions, our results demonstrated at the regional scale that the fungal community Co and Cf were significantly positively correlated with latitude (Figure 7). In other words, there was a high fungal concentration and Co in the high latitudinal region where temperature was relatively lower, which indicates that some fungi (e.g., *Tremellomycetes* mention above) are more adapted to survival in cold areas. Staddon and colleagues have reported that microbial functional diversity in soil was negatively correlated to latitude (Staddon et al., 1998). A similar correlation was observed in the present study. The response model displayed that the fungal community dynamics were positively corelated with the altitude, and there was an optimal altitude (ca. 230 m) for the fungal community habitat (Figure 7), at which fungi have the highest biomass (Cf) and community organization (Co). Wu and co-workers have also found that fungal community changed with altitude in the Changjiang River wetlands (Wu et al., 2013). Such trends could be the result of uplift of altitude, which affects the development of drainage systems then in turn greatly influences the biogeography of microbial assemblages (Qi et al., 2007).

In addition, the response model also revealed that different nutrient elements have different effects in terms of Co, Dy, and Cf. The results showed that organic matter (OM) and total nitrogen (TN) only affected fungal biomass (Cf), while nitrate nitrogen (NON), total phosphorus (TP), and quickly available phosphorus (QAP) only had an impact on fungal community structure (Co/Dy) (Figure 7). In contrast, ammonia nitrogen (AON), Eh, and pH have influenced both fungal biomass and community structure. It has been reported that nutrient elements (e.g., C and N) greatly impact microbial biomass and community structures in soil (Van Horn et al., 2013; Yu et al., 2013), and formation of nitrogen (total nitrogen, ammonium nitrogen and nitrate nitrogen) has an opposite effect on bacterial abundance in lake sediment (Song et al., 2010). In the current study, we demonstrated that the fungal biomass is mainly related to the total nutrient content (OM and TN), while the community structure and composition depends on the available formation of elements in the river sediment. Moreover, the results showed that phosphorus has a negative effect on the fungal community organization, which suggests that phosphorus would limit the distribution of predominant fungi in sediment.

In summary, although the river system is self-connected and geographically variable, our data showed that the fungal community has clear geographical patterns in the Songhua River catchment. Compared to the mainstream, the tributaries have a high fungal community organization and culturable fungal concentration, but possess lower community dynamics. The phylogenetic analysis of DGGE bands showed that ascomycota and basidiomycota were the predominant communities in the Songhua River catchment. Moreover, the variation of fungal community structure was primarily dependent on longitude and fungal biomass was mainly related to the total nutrient content, while community structure and composition were affected by the available formation of elements in the river sediment. The results should contribute toward a greater understanding of fungal community distribution in a temperate zone river wetland on a catchment scale.

### Conflict of interest statement

The authors declare that the research was conducted in the absence of any commercial or financial relationships that could be construed as a potential conflict of interest.
