# Supplementary material for: Distribution and diversity of fungi in freshwater sediments on a river catchment scale
Source: Front Microbiol. 2015 Apr 21;6:329. doi: 10.3389/fmicb.2015.00329 (PMC4404825; doi:10.3389/fmicb.2015.00329)
Supplement: Supplementary file 1 [file Table1.DOC]

Supporting information

Appendix S1 Putative taxonomic affinities of DGGE sequenced bands

| DGGE Sequenced bands | Sequence length(bp) | Most taxonomic affinity | Query coveraye(%) | Max ident(%) | Relative Proportion in sample(%) | Taxonomic group | Accession Code |
| --- | --- | --- | --- | --- | --- | --- | --- |
| N1-1 | 187 | *Mortierella cf. hyaline* TEA059 ([AY157495.1](http://www.ncbi.nlm.nih.gov/nucleotide/37196414?report=genbank&log$=nucltop&blast_rank=11&RID=M3UAT5Z8012)) | 100 | 100 | 34.9 | *Mortierella.* | LM655253 |
| N3-1 | 199 | *Pseudeurotium* sp. BEA-2010 ([HM589244.1](http://www.ncbi.nlm.nih.gov/nucleotide/306850946?report=genbank&log$=nucltop&blast_rank=2&RID=M3YJ6FKC012)) | 100 | 100 | 11.9 | *Pseudeurotium.* | LM655254 |
| N7-1 | 218 | *Hypocreales* sp. NIOCC F30 ([EU729707.1](http://www.ncbi.nlm.nih.gov/nucleotide/194173340?report=genbank&log$=nucltop&blast_rank=18&RID=M4077UFU015)) | 98 | 99 | 11.3 | *Hypocreales;* | LM655255 |
| N9-1 | 215 | Uncultured *Ascomycota* ([FJ553111.1](http://www.ncbi.nlm.nih.gov/nucleotide/219812897?report=genbank&log$=nucltop&blast_rank=2&RID=M42RA3N6015)) | 100 | 96 | 12.7 | *Ascomycota* | LM655256 |
| N9-2 | 247 | *Phanerochaete chrysosporium* ([HQ188435.1](http://www.ncbi.nlm.nih.gov/nucleotide/307950947?report=genbank&log$=nucltop&blast_rank=1&RID=M4F4S6W7012)*)* | 100 | 99 | 7.7 | *Phanerochaete.* | LM655257 |
| N11-1 | 253 | Uncultured *Chytridiomycota (*[HQ191391.1](http://www.ncbi.nlm.nih.gov/nucleotide/311408970?report=genbank&log$=nucltop&blast_rank=1&RID=M4G4YNC2012)*)* | 43 | 87 | 9.9 | *Chytridiomycota;* | LM655258 |
| N12-1 | 173 | *Cladosporium cladosporioides* ([AB693799.1](http://www.ncbi.nlm.nih.gov/nucleotide/374346088?report=genbank&log$=nucltop&blast_rank=1&RID=R8EY0RPA01S)) | 100 | 99 | 14.6 | *Cladosporium.* | LM655259 |
| N15-1 | 209 | *Mrakia sp.* KLP-2011d ([JF720029.1](http://www.ncbi.nlm.nih.gov/nucleotide/335355492?report=genbank&log$=nucltop&blast_rank=1&RID=M4K5PP1401R)) | 100 | 99 | 15.6 | *Mrakia.* | LM655260 |
| N17-1 | 189 | *Mrakia* sp. KLP-2011d ([JF720029.1](http://www.ncbi.nlm.nih.gov/nucleotide/335355492?report=genbank&log$=nucltop&blast_rank=1&RID=R8R149T801N)) | 100 | 99 | 10.7 | *Mrakia.* | LM655261 |
| N18-1 | 269 | *Mrakia sp* KLP-2011d ([JF720029.1](http://www.ncbi.nlm.nih.gov/nucleotide/335355492?report=genbank&log$=nucltop&blast_rank=1&RID=M6C2H8CD012)) | 75 | 99 | 14.6 | *Mrakia.* | LM655262 |
| N20-1 | 259 | *Mortierella minutissima* ([EU484297.1](http://www.ncbi.nlm.nih.gov/nucleotide/171467218?report=genbank&log$=nucltop&blast_rank=9&RID=M6P32UPS01R)) | 72 | 94 | 17.7 | *Mortierella.* | LM655263 |
| N24-1 | 197 | Uncultured eukaryote ([EU003080.1](http://www.ncbi.nlm.nih.gov/nucleotide/154082256?report=genbank&log$=nucltop&blast_rank=1&RID=M9D6BNVB01S)*)* | 92 | 77 | 11.9 | Unidentified | LM655264 |
| N25-1 | 230 | *Candida* sp. MCCF-101 ([FJ652052.1](http://www.ncbi.nlm.nih.gov/nucleotide/223551489?report=genbank&log$=nucltop&blast_rank=24&RID=M9MVT43V01R)) | 79 | 89 | 19.2 | *Candida.* | LM655265 |
| N25-2 | 215 | *Cochliobolus kusanoi* ([JN943393.1](http://www.ncbi.nlm.nih.gov/nucleotide/363544553?report=genbank&log$=nucltop&blast_rank=3&RID=M9NCWRCX01R)) | 97 | 99 | 15.0 | *Pleosporineae* | LM655266 |
| N25-3 | 220 | Uncultured fungus ([FN397288.1](http://www.ncbi.nlm.nih.gov/nucleotide/256855775?report=genbank&log$=nucltop&blast_rank=3&RID=M9R1TCWP013)*)* | 100 | 95 | 17.6 | Unidentified | LM655267 |
| N27-1 | 221 | *Simplicillium sp.* MS-2011-F21 ([HE608791.1](http://www.ncbi.nlm.nih.gov/nucleotide/355321689?report=genbank&log$=nucltop&blast_rank=2&RID=M9S4K04S01S)) | 100 | 99 | 8.3 | *Simplicillium.* | LM655268 |
| N34-1 | 177 | *Mrakiella aquatic* ([AY052488.1](http://www.ncbi.nlm.nih.gov/nucleotide/16209526?report=genbank&log$=nucltop&blast_rank=4&RID=M9TRPV9U01R)) | 100 | 100 | 20.9 | *Mrakiella.* | LM655269 |
| N34-2 | 193 | *Fusarium solani* ([FR691775.1](http://www.ncbi.nlm.nih.gov/nucleotide/319801168?report=genbank&log$=nucltop&blast_rank=1&RID=NBHWD7DP01N)) | 100 | 100 | 38.6 | *Hypocreales* | LM655270 |
| N35-1 | 231 | Uncultured fungus ([HQ388460.1](http://www.ncbi.nlm.nih.gov/nucleotide/312843799?report=genbank&log$=nucltop&blast_rank=1&RID=M3SBAWMS015)) | 35 | 97 | 8.9 | Unidentified | LM655271 |
| N35-2 | 264 | *Mrakia sp*.KLP-2011d ([JF720029.1](http://www.ncbi.nlm.nih.gov/nucleotide/335355492?report=genbank&log$=nucltop&blast_rank=1&RID=MEHHW15F012)) | 78 | 98 | 20.8 | *Mrakia.* | LM655272 |
| N43-1 | 193 | *Mortierella minutissima* ([EU484297.1](http://www.ncbi.nlm.nih.gov/nucleotide/171467218?report=genbank&log$=nucltop&blast_rank=9&RID=MEX11GJJ012)) | 100 | 94 | 21.8 | *Mortierella.* | LM655273 |
| N43-2 | 201 | Uncultured fungus ([EU825622.1](http://www.ncbi.nlm.nih.gov/nucleotide/194354250?report=genbank&log$=nucltop&blast_rank=1&RID=M3N7NKE401S)*)* | 99 | 98 | 24.2 | Unidentified | LM655274 |
| N44-1 | 187 | Uncultured eukaryote ([EU003080.1](http://www.ncbi.nlm.nih.gov/nucleotide/154082256?report=genbank&log$=nucltop&blast_rank=1&RID=MF22K7JE016)) | 100 | 81 | 15.2 | Unidentified | LM655275 |
| N46-1 | 227 | Uncultured fungus ([FJ687268.2](http://www.ncbi.nlm.nih.gov/nucleotide/315305424?report=genbank&log$=nucltop&blast_rank=1&RID=R94D6WZ101S)) | 100 | 98 | 17.6 | Unidentified | LM655276 |
| N46-2 | 231 | *Candida* sp. MCCF-101 ([FJ652052.1](http://www.ncbi.nlm.nih.gov/nucleotide/223551489?report=genbank&log$=nucltop&blast_rank=24&RID=MF2YP1HV013)) | 80 | 89 | 5.9 | *Candida.* | LM655277 |
| N47-1 | 178 | *Phoma* sp. R79-10 ([AB693778.1](http://www.ncbi.nlm.nih.gov/nucleotide/373938213?report=genbank&log$=nucltop&blast_rank=1&RID=MF4JJCR9013)) | 100 | 100 | 10.3 | *Pleosporineae* | LM655278 |
| N47-2 | 200 | *Aspergillus versicolor* ([JN942862.1](http://www.ncbi.nlm.nih.gov/nucleotide/358001535?report=genbank&log$=nucltop&blast_rank=1&RID=R94M4NA401S)*)* | 98 | 100 | 13.8 | *Pezizomycotina* | LM655279 |
| N48-1 | 177 | Uncultured fungus ([FJ626913.1](http://www.ncbi.nlm.nih.gov/nucleotide/223453053?report=genbank&log$=nucltop&blast_rank=1&RID=M3RGRCSY012)*)* | 49 | 86 | 19.2 | Unidentified | LM655280 |
| N51-1 | 220 | Uncultured fungus ([FN397288.1](http://www.ncbi.nlm.nih.gov/nucleotide/256855775?report=genbank&log$=nucltop&blast_rank=3&RID=MGZ24TCR01N)*)* | 100 | 96 | 32.4 | Unidentified | LM655281 |
| N54-1 | 215 | *Simplicillium sp.* MS-2011-F21 ([HE608791.1](http://www.ncbi.nlm.nih.gov/nucleotide/355321689?report=genbank&log$=nucltop&blast_rank=2&RID=MH03EFWH016)*)* | 100 | 99 | 11.3 | *Simplicillium.* | LM655282 |
| N58-1 | 201 | Uncultured fungus ([FJ528686.1](http://www.ncbi.nlm.nih.gov/nucleotide/257122659?report=genbank&log$=nucltop&blast_rank=1&RID=RRJ6A9G3016)) | 45 | 90 | 10.2 | Unidentified | LM655283 |
| N58-2 | 202 | *Mrakiella aquatic* ([AY052488.1](http://www.ncbi.nlm.nih.gov/nucleotide/16209526?report=genbank&log$=nucltop&blast_rank=3&RID=MH11NAT4016)) | 100 | 100 | 13.4 | *Mrakiella.* | LM655284 |
| N62-1 | 181 | *Aspergillus ochraceus* ([HE608162.1](http://www.ncbi.nlm.nih.gov/nucleotide/371781949?report=genbank&log$=nucltop&blast_rank=1&RID=MHRBHY8X01S)) | 99 | 99 | 9.0 | *Aspergillus.* | LM655285 |
| N71-1 | 173 | *Basidiobolus haptosporus* ([EF392531.1](http://www.ncbi.nlm.nih.gov/nucleotide/145307995?report=genbank&log$=nucltop&blast_rank=3&RID=MHSSDFHP013)) | 100 | 98 | 8.5 | *Basidiobolus.* | LM655286 |
| N72-1 | 213 | *Phanerochaete chrysosporium* ([JN882305.1](http://www.ncbi.nlm.nih.gov/nucleotide/373879651?report=genbank&log$=nucltop&blast_rank=1&RID=MHTHYZGD012)*)* | 100 | 100 | 20.1 | *Phanerochaete* | LM655287 |
| N81-1 | 139 | *Basidiobolus ranarum* ([JN943057.1](http://www.ncbi.nlm.nih.gov/nucleotide/358441792?report=genbank&log$=nucltop&blast_rank=14&RID=MKS84JVG01N)) | 26 | 100 | 16.8 | *Basidiobolus.* | LM655288 |
| N82-1 | 225 | *Wardomyces inflatus*([FJ946485.1](http://www.ncbi.nlm.nih.gov/nucleotide/237860272?report=genbank&log$=nucltop&blast_rank=1&RID=MKT343NH01N)) | 99 | 98 | 16.6 | *Wardomyces.* | LM655289 |
| N82-2 | 171 | *Simplicillium chinense* ([JQ410323.1](http://www.ncbi.nlm.nih.gov/nucleotide/387317105?report=genbank&log$=nucltop&blast_rank=9&RID=BS4P4GHK01R)) | 100 | 99 | 16.7 | *Hypocreales* | LM655290 |
| N85-1 | 210 | *Penicillium terrigenum* ([JN617684.1](http://www.ncbi.nlm.nih.gov/nucleotide/372123140?report=genbank&log$=nucltop&blast_rank=1&RID=MKTR0T8W01N)) | 100 | 100 | 29.2 | *Penicillium.* | LM655291 |
| N89-1 | 171 | Uncultured fungus ([JQ038319.1](http://www.ncbi.nlm.nih.gov/nucleotide/380083065?report=genbank&log$=nucltop&blast_rank=1&RID=R95SBY2A01S)*)* | 100 | 98 | 11.1 | Unidentified | LM655292 |
| N91-1 | 182 | *Mortierella elongate* ([HQ630362.1](http://www.ncbi.nlm.nih.gov/nucleotide/325516935?report=genbank&log$=nucltop&blast_rank=3&RID=MKUU357P01S)) | 72 | 98 | 14.0 | *Mortierella.* | LM655293 |
| N92-1 | 173 | *Phoma tropica* ([JF923821.1](http://www.ncbi.nlm.nih.gov/nucleotide/344222099?report=genbank&log$=nucltop&blast_rank=2&RID=R96NVJXA01N)) | 100 | 95 | 18.7 | *Pleosporales* | LM655294 |
| N95-1 | 187 | *Mortierella cf. hyaline* ([AY157495.1](http://www.ncbi.nlm.nih.gov/nucleotide/37196414?report=genbank&log$=nucltop&blast_rank=15&RID=R96C113D01S)) | 96 | 99 | 13.8 | *Mortierellales* | LM655295 |
| N95-2 | 171 | *Candida viswanathii* ([FJ542757.1](http://www.ncbi.nlm.nih.gov/nucleotide/220683597?report=genbank&log$=nucltop&blast_rank=6&RID=MKYM9EXD01S)*)* | 100 | 100 | 17.2 | *Candida.* | LM655296 |
| N96-1 | 281 | Uncultured fungus ([JF300384.1](http://www.ncbi.nlm.nih.gov/nucleotide/342672989?report=genbank&log$=nucltop&blast_rank=1&RID=MM61MFH401N)) | 29 | 89 | 14.2 | Unidentified | LM655297 |
| N96-2 | 263 | *Arachnomyces glareosus voucher* ([AY624316.1](http://www.ncbi.nlm.nih.gov/nucleotide/54289558?report=genbank&log$=nucltop&blast_rank=2&RID=MM6EF64N012)) | 87 | 93 | 16.3 | *Arachnomyces.* | LM655298 |
| N97-1 | 164 | *Verticillium* sp. 03VT08 ([JX270367.1](http://www.ncbi.nlm.nih.gov/nucleotide/411030901?report=genbank&log$=nucltop&blast_rank=1&RID=BY1T5R08016)) | 100 | 99 | 11.2 | *Hypocreales;* | LM655299 |
| N101-1 | 113 | *Diversispora celata* ([AM713404.1](http://www.ncbi.nlm.nih.gov/nucleotide/224586616?report=genbank&log$=nucltop&blast_rank=1&RID=R97HMUZV01N)) | 100 | 100 | 13.7 | *Glomeromycetes* | LM655300 |
| N101-2 | 212 | *Penicillium terrigenum* ([JN617684.1](http://www.ncbi.nlm.nih.gov/nucleotide/372123140?report=genbank&log$=nucltop&blast_rank=1&RID=MM9D2S4J01N)) | 100 | 100 | 13.4 | *Penicillium.* | LM655301 |
| N101-3 | 238 | *Hypocrea* *rufa* ([AB374534.1](http://www.ncbi.nlm.nih.gov/nucleotide/371919173?report=genbank&log$=nucltop&blast_rank=2&RID=MM9T1VVD01N)) | 100 | 99 | 17.2 | *Hypocrea.* | LM655302 |
| N102-2 | 198 | *Phialophora* sp.( [EU314707.1](http://www.ncbi.nlm.nih.gov/nucleotide/169667539?report=genbank&log$=nucltop&blast_rank=21&RID=MMAAEM9C01N)) | 100 | 97 | 25.3 | *Phialophora.* | LM655303 |
| N102-3 | 187 | *Epicoccum* sp. OUCMBI101237 ([HQ914877.1](http://www.ncbi.nlm.nih.gov/nucleotide/327387909?report=genbank&log$=nucltop&blast_rank=1&RID=R97WRRKG01N)) | 98 | 100 | 14.8 | *Epicoccum.* | LM655304 |
| N103-1 | 201 | Uncultured fungus ([HQ873329.1](http://www.ncbi.nlm.nih.gov/nucleotide/324034764?report=genbank&log$=nucltop&blast_rank=1&RID=R986D09G01N)*)* | 83 | 94 | 24.8 | Unidentified | LM655305 |
| N108-1 | 193 | Uncultured *Oidiodendron* ([FJ553111.1](http://www.ncbi.nlm.nih.gov/nucleotide/219812897?report=genbank&log$=nucltop&blast_rank=1&RID=R987SA9V01N)) | 100 | 98 | 14.1 | *Myxotrichaceae* | LM655306 |
| N112-1 | 189 | *Guehomyces pullulans* ([HQ615695.1](http://www.ncbi.nlm.nih.gov/nucleotide/327554598?report=genbank&log$=nucltop&blast_rank=2&RID=MMAXEV1K012)) | 97 | 100 | 16.2 | *Guehomyces* | LM655307 |
| N115-1 | 209 | *Mrakia* sp.([JF720029.1](http://www.ncbi.nlm.nih.gov/nucleotide/335355492?report=genbank&log$=nucltop&blast_rank=1&RID=MMBAM5BJ012)) | 100 | 99 | 10.9 | *Mrakia.* | LM655308 |
| N115-2 | 203 | *Podospora appendiculata* ([AY999126.1](http://www.ncbi.nlm.nih.gov/nucleotide/67644101?report=genbank&log$=nucltop&blast_rank=1&RID=MMBR4RKK01S)) | 99 | 98 | 14.4 | *Podospora.* | LM655309 |
| N117-1 | 179 | *Chaetomium globosum* ([JN689341.1](http://www.ncbi.nlm.nih.gov/nucleotide/359392749?report=genbank&log$=nucltop&blast_rank=1&RID=MMCCSPBW012)) | 100 | 100 | 14.2 | *Chaetomium.* | LM655310 |

Appendix S2 The variance of each factor explained in RDA

| Variable | LO | TP | OM | QAP | TN | Eh | AON | AL | NON | LA | pH |
| --- | --- | --- | --- | --- | --- | --- | --- | --- | --- | --- | --- |
| Variance explained/% | 19.82 | 11.01 | 11.01 | 11.01 | 11.01 | 8.81 | 8.81 | 8.81 | 6.61 | 2.20 | 0 |
| P-value | 0.002 | 0.002 | 0.002 | 0.002 | 0.004 | 0.004 | 0.002 | 0.002 | 0.01 | 0.076 | 0.266 |
| F-ratio | 11.44 | 8.43 | 7.61 | 10.44 | 8.76 | 5.39 | 5.75 | 6.07 | 4.78 | 2.38 | 1.25 |

Appendix S3 DGGE pattern of partial fungal ITS sequences amplified from the Songhua River catchment sites. Lane numbers correspond to sampling points along the river from downstream to upstream.
